# Soluble ST2 and All-Cause Mortality in Patients with Chronic Obstructive Pulmonary Disease—A 10-Year Cohort Study

**DOI:** 10.3390/jcm11010056

**Published:** 2021-12-23

**Authors:** Matthias H. Urban, Stefan Stojkovic, Svitlana Demyanets, Christian Hengstenberg, Arschang Valipour, Johann Wojta, Otto C. Burghuber

**Affiliations:** 1 Department of Internal and Respiratory Medicine and Karl-Landsteiner-Institute for Lung Research and Pulmonary Oncology, Klinik Floridsdorf, 1210 Vienna, Austria; matthias.urban@gesundheitsverbund.at (M.H.U.); arschang.valipour@gesundheitsverbund.at (A.V.); 2 Ludwig Boltzmann Institute for Lung Health, 1140 Vienna, Austria; otto.burghuber@extern.gesundheitsverbund.at; 3 Department of Internal Medicine II, Division of Cardiology, Medical University of Vienna, 1090 Vienna, Austria; stefan.stojkovic@meduniwien.ac.at (S.S.); christian.hengstenberg@meduniwien.ac.at (C.H.); 4 Ludwig Boltzmann Institute for Cardiovascular Research, 1090 Vienna, Austria; 5 Department of Laboratory Medicine, Medical University of Vienna, 1090 Vienna, Austria; svitlana.demyanets@meduniwien.ac.at; 6 Core Facilities, Medical University of Vienna, 1090 Vienna, Austria; 7 Medical School, Sigmund Freud University, 1020 Vienna, Austria

**Keywords:** chronic obstructive pulmonary disease, inflammation, prognosis, biomarker, sST2

## Abstract

Chronic obstructive pulmonary disease (COPD) is an inflammatory condition with constantly increasing mortality rates. Interleukin (IL)-33 and its decoy receptor, soluble suppression of tumorigenicity 2 (sST2), play a central role in the inflammatory response during infection. sST2 was suggested as a factor in the pathogenesis of COPD and emerged as a predictor of mortality in other non-communicable diseases. The role of sST2 as a predictor of mortality remains unclear in COPD yet. In this cohort study, we measured circulating concentrations of IL-33 and sST2 in the serum of patients with stable COPD (*n* = 59), patients with acute exacerbation of COPD (*n* = 29) and smoking (*n* = 20) and non-smoking controls (*n* = 20), using commercially available ELISAs, and investigated the prognostic role of sST2 in stable COPD. sST2 levels were significantly higher in COPD patients and smokers compared with non-smoking controls. We identified systolic blood pressure, forced expiratory volume in 1 s (FEV1% predicted), neutrophil count, lactate dehydrogenase and pack-years index as independent predictors of sST2 levels. During a median follow-up time of 10.6 years, 28 patients (47.5%) died. sST2 was an independent predictor of all-cause mortality in patients with COPD with a hazard ratio of 2.9 (95% CI 1.1–8.4, *p* = 0.035) per one standard deviation after adjustment for age, sex, pack-years, FEV1% predicted and C-reactive protein (CRP). sST2 concentrations are associated with severity of disease and long-term outcome in patients with COPD.

## 1. Introduction

Chronic obstructive pulmonary disease (COPD) is associated with a chronic inflammatory process of the lungs. In contrast to other non-communicable diseases, COPD-related mortality is constantly increasing over decades [1,2]. Airflow limitation is the central pathophysiologic feature and an indicator of disease severity in COPD. However, its predictive value for mortality is limited, especially at an individual level [3].

Well-established predictors of mortality in COPD comprise exacerbations [4,5], infections with inflammatory host response [6,7,8] and, most notably, comorbidities [9,10]. Both inflammatory host response [11] and comorbidities such as heart failure (HF) and coronary artery disease (CAD) revealed augmented expressions of interleukin (IL)-33 and soluble suppression of tumorigenicity 2 (sST2) [12,13].

Circulating levels of sST2 correlate with cardiac remodeling and fibrosis and are strongly predictive of death in patients with HF [14,15,16] and CAD [17,18]. Furthermore, sST2 was identified as a predictor for mortality in chronic kidney disease [19,20], hepatic failure [21,22] and critically ill patients [23,24,25].

Recent studies suggested a critical role for the IL-33/sST2 pathway in the pathogenesis of COPD [26,27]. IL-33 is supposed to trigger adaptive immune responses and thereby mediate cigarette-smoke-induced tissue damage in the lungs [28,29]. Smoking increases IL-33 expression and alters ST2 receptor expression, thus facilitating IL-6- and IL-8-mediated inflammatory response [29,30]. COPD patients have elevated serum levels of IL-33 and sST2 [31], and IL-33 expression is also increased in the lungs of stable COPD patients, but serum levels were reduced during acute exacerbation [26,27,31]. To our knowledge, no study has investigated sST2 as a predictor of long-term mortality in COPD yet.

In this study, we aimed to compare levels of circulating sST2 and IL-33 between COPD patients and controls and to assess the prognostic value of sST2 on long-term outcomes in COPD. We hypothesized that sST2 independently predicts all-cause mortality in patients with COPD.

## 2. Materials and Methods

### 2.1. Study Design and Participants

For this prospective cohort study, patients with COPD were recruited via the outpatient clinic of the Otto Wagner Hospital, Vienna. A sub-sample of COPD patients was enrolled during an acute exacerbation. Inclusion criteria comprised age above 40 years, a smoking history of at least 20 pack-years and spirometrically confirmed airflow limitation. Stable COPD was defined as no acute exacerbation for 8 weeks prior to inclusion. Exacerbations were characterized by a worsening of COPD-related symptoms requiring escalation of therapy including systemic corticosteroids and/or antibiotic therapy [32]. Exclusion criteria covered the regular use of oral corticosteroids, asthma, CAD, HF, diabetes, autoimmune diseases, chronic renal failure, liver diseases or critical illness. 

Control groups comprised smoking and non-smoking individuals at a ratio of 3 (COPD):1 (smoking):1(non-smoking). Controls were matched for age and had to have no signs of airflow limitation on spirometry. Recruitment lasted from October 2005 to August 2006, and the follow-up data were collected in February 2017 via the registry of the Austrian government (Statistics Austria). The study was approved by the Ethics Committee of the Vienna City Council (EK-number: EK05-150-1205). Written informed consent was obtained from each participant.

### 2.2. Subject Characteristics and Study Endpoints

The primary endpoint was time to death of any cause in patients with stable COPD. Participants’ vital status was obtained from Statistics Austria. Physical examination was performed for each participant, including body mass index (BMI), blood pressure, heart rate and 6 min walking test (6-MWT). Lung function was tested by means of spirometry and body plethysmography following the standardization of the European Respiratory Society and the American Thoracic Society [33]. Venous blood samples were taken in the morning in a fasting state from participants’ antecubital fossa. Whole blood was centrifuged (at 2500 rpm for 10 min), and serum was stored for subsequent analysis in a lab freezer at −80 °C.

### 2.3. IL-33 and sST2 Measurement

sST2 was quantified using the human ST2/IL-1 R4 DuoSet^®^ ELISA Kit (R&D Systems, Minneapolis, MN, USA) [12,14,25].

Circulating levels of IL-33 were measured using the Human IL-33 DuoSet^®^ ELISA (R&D Systems, Minneapolis, MN, USA) as described by our group previously [14,25,34].

### 2.4. Statistical Analysis

Categorical variables are summarized as counts and percentages and are compared by the χ^2^-test or by Fisher’s exact test as appropriate. Continuous variables are expressed as median and interquartile range (IQR) and compared by one-way ANOVA with Dunnett’s T3 post hoc analysis or the Kruskal–Wallis test in case of non-normal distribution. For paired measurements, the Wilcoxon matched-pairs signed-ranks test was used. Univariate correlation was calculated by means of Spearman’s rank-order correlation coefficient. Multivariate linear regression was conducted using stepwise selection of significant univariate predictors of sST2. Univariate and multivariable Cox proportional hazard regression models were fit to assess whether sST2 could significantly predict all-cause mortality. Hazard ratios (HRs) are given as HR per increase of one standard deviation (HR per 1-SD). Due to their skewed distributions, log2-transformed values of sST2 were used within all regression models. Optimal cut-off values for sST2 were calculated using Cut-off Finder’s significance of correlation with survival variable (http://molpath.charite.de/cutoff, accessed 14 June 2019) [35] and used to construct Kaplan–Meier survival plots. Harrell’s C-statistic was applied to evaluate predictive power of sST2 when added to established clinical risk factors. Two-sided *p*-values of ≤0.05 indicated statistical significance. SPSS 22.0 (IBM Corporation, Armonk, NY, USA) and STATA version 12 (StataCorp LLC., College Station, TX, USA) were used for all statistical analyses. Data are reported in accordance with the STROBE Statement (Strengthening the Reporting of Observational Studies in Epidemiology) [36].

## 3. Results

### 3.1. Subject Characteristics

The study groups were comparable in terms of age, sex and BMI (Table 1). Statistically significant differences were observed in heart rate, CRP and neutrophil count with the highest levels found during acute exacerbation (Table 1). Levels of NT-proBNP were under 80 ng/L in all study groups.

### 3.2. IL-33, sST2 and Determinants of COPD

**Figure 1:**
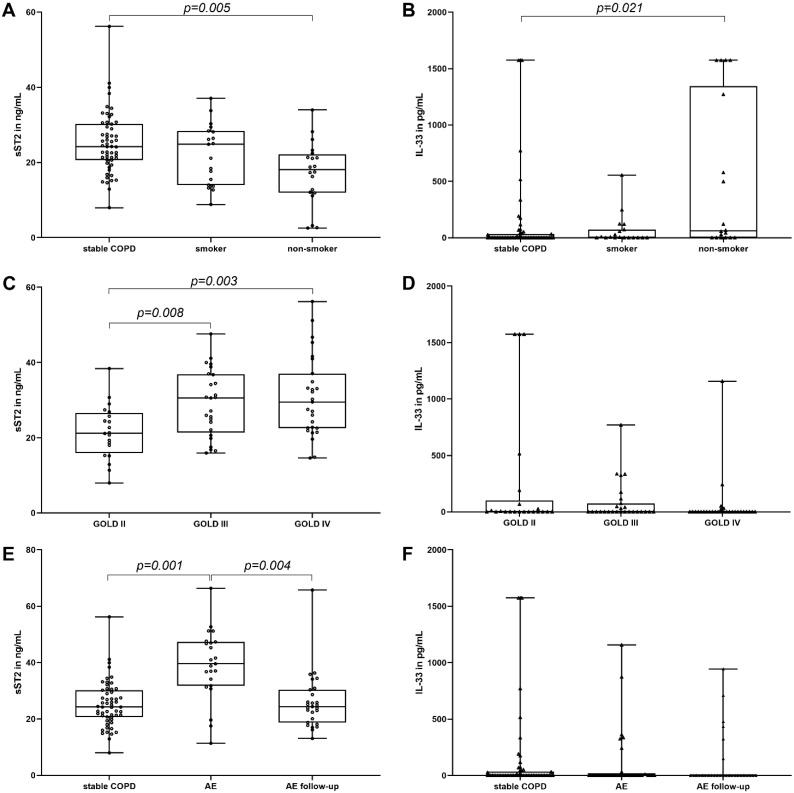
IL-33 and soluble ST2 levels in non-smokers, smokers, patients with stable COPD (panels **A** and **B**), GOLD stage 2–4 (panels **C** and **D**), and exacerbated COPD (panels **E** and **F**). Circulating IL-33 and sST2 were measured as described in the Methods section. Concentrations are depicted as median with interquartile range and given in ng/mL (sST2) and pg/mL (IL-33). Groups were compared using one-way ANOVA as described in Methods section. *p*-values < 0.05 were considered significant. AE, acute exacerbation.

Levels of sST2 were overall higher in COPD versus non-smoking controls (24.2 ng/mL, interquartile range (IQR) 20.6–30.2 vs. 18.1 ng/mL, IQR 11.9–22.2, *p* = 0.005, Figure 1A) and increased with increasing levels by GOLD stage (Figure 1C). Furthermore, acute exacerbated COPD patients showed elevated sST2 levels compared to stable COPD (39.59 ng/mL, IQR 31.77–44.33 vs. 24.2 ng/mL, IQR 20.6–30.2, *p* = 0.001, Figure 1E). Following acute exacerbation, circulating sST2 concentration returns to baseline levels (24.36 ng/mL, IQR 18.8–30.3, *p* = 0.004, as compared to acute exacerbation, Figure 1E). IL-33 was higher in non-smokers (61.09 pg/mL, IQR 0–1346.06) as compared to stable COPD (0 pg/mL, IQR 0–33.99, *p* = 0.021), and there was a tendency, as compared to smokers, but it did not reach statistical significance (4.08 pg/mL, IQR 0–72.53, *p* = 0.860, Figure 1B). Patients with COPD had comparable IL-33 levels regardless of disease severity (GOLD 2: 0 pg/mL, IQR 0–99.12 vs. GOLD 3: 0 pg/mL, IQR 0–74.67 vs. GOLD 4: 0 pg/mL, IQR 0–0.55, *p* = 0.185, Figure 1D). IL-33 was below detection limit in 36 (61%) patients with stable COPD and in 21 (72%) exacerbated patients.

**Figure 2:**
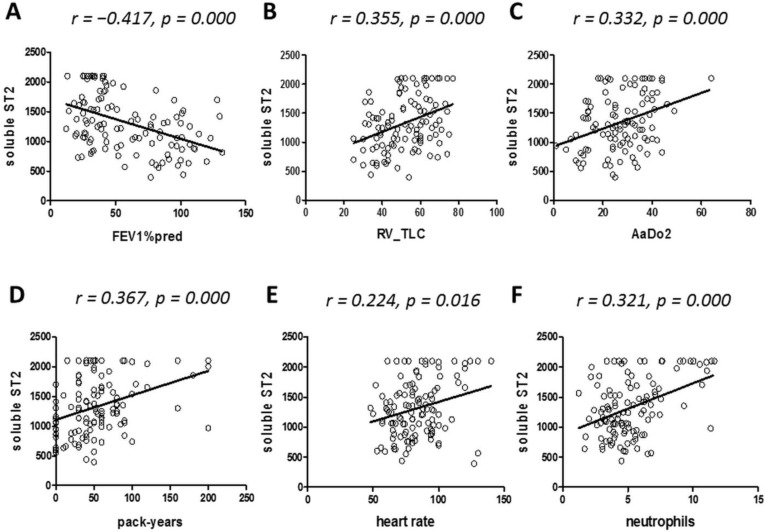
Determinants of soluble ST2 in COPD patients and healthy controls. Univariate correlations between soluble ST2 (in ng/mL) and FEV1% predicted (panel **A**), RV and TLC (in L/L) (panel **B**), AaDO2 (in mmHg) (panel **C**), pack-years (panel **D**), heart rate (in bpm) (panel **E**) and neutrophil count (in G/L) (panel **F**) in the entire study sample (*n* = 118). Circulating sST2 was measured as described in the Methods section; *p*-values < 0.05 were considered significant.

FEV1% predicted was inversely correlated with sST2 in the total sample (Figure 2A). sST2 showed a significant univariate association to total lung capacity (Figure 2B), alveolar–arterial oxygen difference (Figure 2C), pack-years (Figure 2D) and heart rate (Figure 2E). sST2 significantly correlated with neutrophil count in our study sample. A detailed list of univariate correlations between sST2 and selected variables in each study group is given in Supplementary Table S1. In a multivariate analysis, we identified systolic blood pressure, FEV1% predicted, pack-years, neutrophils and LDH as independent predictors of sST2 with 68.4% variance explained by the model (R^2^ = 0.468) (Table 2).

### 3.3. sST2 Predicts Mortality in COPD

During the median follow-up time of 10.6 years, 28 patients (47.5%) died. Of these, 20 patients (71.4%) died of COPD-related respiratory failure. Four patients (14.3%) died from pulmonary malignancy, two patients (7.1%) died from cardiovascular death and two patients (7.1%) from liver cirrhosis and liver failure.

Cox proportional hazard regression models were fit to assess the predictive values of sST2 for all-cause mortality. In univariable Cox regression, sST2 was a strong predictor of mortality with HR per 1-SD 3.9 (95% confidence interval (CI) 1.7–9.4, *p* = 0.002, Table 3). sST2 independently predicted all-cause mortality after adjustment for age and sex with HR per 1-SD 3.9 (95% CI 1.4–10.9, *p* = 0.007). After further adjustment for pack-years, FEV1% predicted and CRP, sST2 remained a significant independent predictor of all-cause mortality with an adjusted HR per 1-SD 2.9 (95% CI 1.1–8.4, *p* = 0.035).

**Figure 3:**
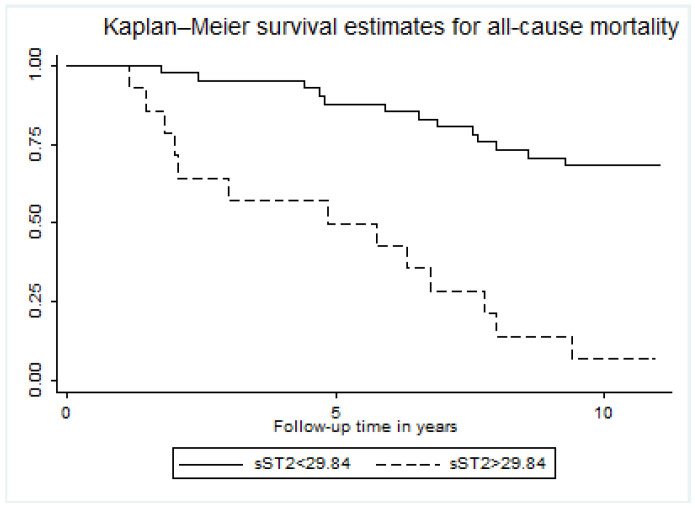
Kaplan–Meyer survival estimates for all-cause mortality in stable COPD stratified according to circulating sST2 levels. Circulating sST2 was measured as described in the Methods section. Survival in groups according to baseline sST2 is depicted. The group with circulating sST2 above the cut-off is indicated by the dotted line, and the full line indicates the group with sST2 below the cut-off.

**Figure 4:**
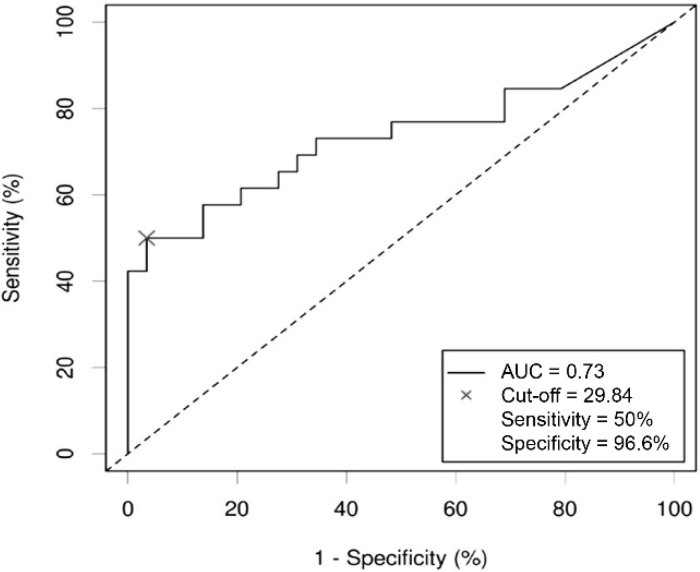
Receiver operating characteristic (ROC) curve for all-cause mortality for sST2 in stable COPD. The prognostic utility of circulating sST2 for outcome in patients with stable COPD. sST2 predicted all-cause mortality with 96.6% specificity and 50% sensitivity and an area under the curve (AUC) of 0.73. Optimal cut-off value for sST2 was calculated and circulating sST2 was measured as described in Methods section.

sST2 was above the calculated threshold of 29.84 ng/mL in 14 COPD patients (23.7%). In the Kaplan–Meier survival plots, sST2 above the cut-off was associated with higher mortality rates as compared to sST2 below the cut-off (log-rank: *p* < 0.001, Figure 3). sST2 predicted mortality with 96.6% specificity and 50% sensitivity and an AUC of 0.73 (Figure 4). Furthermore, when sST2 was added to a model with age, sex, pack-years, FEV1% predicted and CRP, the Harrell’s C-index AUC for prediction of all-cause mortality improved from 0.69 (95% CI 0.59–0.80) to 0.79 (95% CI 0.71–0.87, *p* = 0.036, Table 4).

## 4. Discussion

In the present study, we investigated circulating IL-33 and sST2 in patients with COPD and healthy controls. In COPD, we found significantly increased levels of sST2 and lower levels of IL-33 when compared with healthy controls. Furthermore, sST2 levels increased with disease severity and in acute exacerbation of COPD and showed significant correlations with functional parameters of the disease. We could show for the first time that sST2 is a strong, independent predictor of long-term, all-cause mortality in COPD and improves risk stratification when combined with established clinical risk factors in this group of patients.

IL-33 expression is elevated in the lungs of COPD patients [27,31]. Exposure to cigarette smoke increases IL-33 expression in the lungs and alters ST2 receptor expression, facilitating inflammatory response in acute exacerbation [29]. In mice, cigarette smoke increased IL-33 expression in bronchial endothelial cells, which enhanced systemic inflammation by inducing IL-6 and IL-8 [30]. A previous study by Tang et al. showed lower serum IL-33 during acute exacerbation as compared to stable COPD [26]. Here we showed that circulating IL-33 levels were lower in smokers and COPD patients as compared to non-smokers. On the other hand, circulating sST2 levels were higher in smokers and positively correlated with pack-years, suggesting increased sST2 production after prolonged exposure to cigarette smoke. Furthermore, concentrations of IL-33 were low in COPD patients and did not correlate with disease severity, which is in agreement with previous data [37]. It is noteworthy that IL-33 was undetectable in almost two-thirds of COPD patients and in 72% of patients with an acute exacerbation, making its use as a biomarker in COPD futile. Low or undetectable IL-33 is most likely caused by high circulating levels of sST2. Since sST2 acts as a decoy receptor for IL-33, and IL-33 can induce sST2 production, one could speculate that increased circulating sST2 in COPD reflects increased local IL-33 expression and aggravated inflammatory response in the lungs. This notion is further supported by the correlation of sST2 with inflammatory markers such as CRP or neutrophil count in our study.

We showed here for the first time that sST2 is an independent predictor of all-cause mortality and adds prognostic information on top of clinical risk factors in COPD. However, elevated sST2 levels are also detected after myocardial infarction (MI) and in HF, and sST2 is an established prognostic biomarker in this setting [14,15,17]. To rule out possible confounders, patients with CAD, previous MI and HF were excluded from the study, which was confirmed by low baseline NT-proBNP levels in all study groups. The majority of patients died from respiratory failure, and only two patients died a cardiovascular death. Hence, the prognostic value of sST2 in this study might be specific for COPD and is not influenced by comorbidities such as cardiovascular diseases. These findings are in contrast to observations of Hansell et al., who reported the vast majority of COPD-related deaths being attributable to MI and ischemic heart disease [10]. However, this report may have underestimated COPD-specific mortality due to its retrospective nature of data collection. On the other hand, we need to admit that we ruled out overt cardiovascular diseases during the screening phase, leading to a potential selection bias in subsequent causes of death. Further, our cohort of COPD patients was well-elaborated and documented in terms of their respiratory health status, which might favor an overestimation of COPD-related mortality in subsequent health care services. Divo and colleagues conducted a systematic assessment of comorbidities in COPD, and they identified 12 distinct comorbidities as drivers of mortality [9]. In contrast to malignant disorders and anxiety, cardiovascular diseases revealed a rather modest association with the risk of death in this prospective cohort. A thoroughly evaluated mortality follow-up resulted from the TORCH study [38]. The authors characterized cardiac death as the second leading cause of death in patients with COPD, highlighting the role of comorbidities as a prognostic factor in GOLD.

This study has several limitations. First, due to the small sample size, over-adjustment is possible in the multivariable Cox regression. However, the event rate of over 40% and a sufficiently long follow-up period should overcome statistical concerns. We did not categorize individuals with COPD according to the GOLD ABCD scheme, as the resulting group size would have undercut sufficient statistical power. The sample size resulted from a highly restrictive recruitment phase, which comprised the screening of over 1000 participants. We aimed to achieve a highly selected sample without relevant cardiovascular risk factors or overt cardiovascular diseases, which then again improved the internal validity of the present study. Second, the fact that IL-33 was undetectable in a large proportion of our sample might be a consequence of false-negative test results. According to the information provided by the manufacturer of the ELISA used here for IL-33 determination, a recombinant human ST2/Fc-chimera does not cross-react but does interfere at concentrations >12.5 ng/mL. However, such interference cannot be ruled out, as we in our study detected 2–4× higher concentrations of sST2 in patients with stable GOLD stage III and IV as well as in patients with acute exacerbations of COPD. These observations confirm previous results [37] and can be deduced from high levels of decoying sST2 in our COPD population. An underlying causality in this circumstance has to remain speculative due to the hypothesis-generating nature of our study. Finally, the exclusion of comorbidities, especially cardiovascular diseases, might affect the external validity of our mortality data by means of an unrepresentatively low incidence of fatal cardiovascular events. However, the role of sST2 as a predictor of mortality in HF and CAD is well-known, and our stringent exclusion of cardiovascular diseases hence strengthens the interpretation of COPD-related mortality.

## 5. Conclusions

In conclusion, COPD patients revealed higher circulating levels of sST2 and reduced levels of IL-33. sST2 concentration appears to increase with a higher degree of airflow obstruction and peaks during acute exacerbations of COPD. sST2 emerged as an independent prognostic marker in COPD that could be used for improved risk stratification of this group of patients.

## Figures and Tables

**Table 1:** Baseline clinical characteristics of the study population.

Baseline Characteristics	Non-Smoker (*n* = 20)	Smoker (*n* = 20)	Stable COPD (*n* = 59)	AE COPD (*n* = 29)	*p*-Value *
Demographics					
Age, years	67 (56–74)	59 (54–65)	59 (61–69)	64 (59–70)	0.375
Male sex, n (%)	8 (40)	6 (30)	31 (53)	21 (72)	0.125
BMI, kg/m^2^	25 (23–28)	26 (24–29)	25 (22–27)	25 (21–28)	0.442
Active smoking	0 (0)	20 (100)	24 (41)	10 (35)	<0.001
Pack-years	0 (0–0)	30 (20–58)	54 (40–80)	53 (43–87)	<0.001
Functional parameters					
FEV1, %predicted	101 (85–110)	97 (90–105)	38 (26–56)	34 (29–42)	<0.001
TLC, L	5.5 (4.9–6.1)	5.5 (4.8–6.8)	6.8 (5.9–7.8)	7.0 (6.0–8.5)	<0.001
RV/TLC ratio	39 (36–46)	35 (32–40)	60 (53–66)	60 (51–69)	<0.001
AaDO2, mmHg	22 (11–25)	22 (12–24)	33 (28–38)	33 (21–40)	<0.001
6MWT, meters	561 (462–660)	594 (495–594)	363 (289–528)	330 (238–462)	<0.001
Hemodynamic parameters					
Systolic BP, mmHg	130 (120–130)	115 (110–130)	123 (115–140)	135 (120–140)	0.065
Diastolic BP, mmHg	75 (70–80)	73 (70–80)	80 (70–80)	80 (70–80)	0.396
Heart rate, bpm	71 (65–86)	60 (62–81)	85 (77–96)	87 (77–101)	0.001
Laboratory					
CRP, mg/L	2.0 (1.0–4.0)	2.0 (1.0–4.0)	3.0 (2.0–8.0)	7.0 (2.0–28.0)	0.001
NT-proBNP, ng/L	76 (36–177)	75 (45–166)	77 (47–155)		0.819
Creatinine, mg/dL	0.79 (0.68–0.94)	0.80 (0.68–0.93)	0.76 (0.65–0.88)	0.8 (0.64–1.0)	0.889
Hemoglobin, g/dL	15 (13–15)	15 (14–15)	14 (14–16)	14 (13–16)	0.520
Neutrophils, G/L	4.2 (3.1–5.4)	4.0 (3.5–5.1)	4.7 (3.6–5.8)	7.6 (4.5–10)	<0.001
Platelets, G/L	247 (216–294)	265 (240–291)	250 (223–305)	282 (224–315)	0.795
Biomarkers					
IL-33, pg/mL	61.1 (0–1346)	4.1 (0–73)	0 (0–34)	0 (0–23)	0.002
sST2, ng/mL	18 (12–22)	25 (14–28)	24 (21–30)	37 (31–44)	<0.001

* *p*-values were calculated by ANOVA and refer to the difference between at least two of the study groups; COPD, chronic obstructive pulmonary disease; AE COPD, acute exacerbation of chronic obstructive pulmonary disease; BMI, body mass index; FEV1, % pred., forced expiratory volume in 1 s percentage predicted; TLC, total lung capacity; RV/TLC ratio, residual volume/total lung capacity; AaDO2, alveolar–arterial oxygen difference; 6MWT, 6 min walk test; BP, blood pressure; CRP, C-reactive protein; NT-proBNP, N-terminal pro-brain natriuretic peptide; HbA1c, glycated hemoglobin; IL-33, Interleukin-33; sST2, soluble ST2.

**Table 2:** Multivariate linear regression analysis with soluble ST2 as dependent variable in the total sample (R = 0.684, R^2^ = 0.468, F = 16.88, *p* = 0.000).

Variable	B	SE	Standardize ß	t	*p*-Value
constant	−275.789	391.995		−0.704	0.483
Systolic BP, mmHg	6.062	2.720	0.173	2.229	0.028
FEV1, % predicted	−2.681	1.285	−0.190	−2.086	0.040
Neutrophils, G/L	52.812	15.901	0.269	3.321	0.001
LDH, U/L	3.078	1.002	0.240	3.073	0.003
Pack-years	3.243	0.881	0.318	3.681	0.000

BP, blood pressure; FEV1, % pred., forced expiratory volume in 1 s percentage predicted; LDH, lactate dehydrogenase.

**Table 3:** Prognostic value of soluble ST2 for all-cause mortality in patients with COPD.

	HR per 1-SD	95% CI	*p*-Value
Univariable			
sST2	3.9	1.7–9.4	0.002
Multivariable model 1			
sST2	3.9	1.4–10.9	0.007
Multivariable model 2			
sST2	2.9	1.1–8.4	0.035

Model 1: adjusted for age and sex; Model 2: Model 1 plus pack-years, FEV1 % predicted and CRP; HR per 1-SD, hazard ratio per one increase of standard deviation; CI, confidence interval.

**Table 4:** sST2 adds prognostic information on top of clinical risk factors in patients with COPD.

All-Cause Mortality			
	Harrell’s C-Index	95% CI	*p*-Value
Multivariable model	0.69	0.59–0.80	
Multivariable model and sST2	0.79	0.71–0.87	0.036 *

Multivariable model: adjusted for age and sex, pack-years, FEV1 % predicted and CRP; * as compared to multivariable model; CI, confidence interval.

## Data Availability

The data presented in this study are available on request from the corresponding author.

## Supplementary Materials

The following are available online at https://www.mdpi.com/article/10.3390/jcm11010056/s1, Table S1: Univariate correlations between sST2 and selected variables in the total sample, in patients with stable COPD and exacerbated COPD.
