# Supplementary material for: Soluble ST2 and All-Cause Mortality in Patients with Chronic Obstructive Pulmonary Disease—A 10-Year Cohort Study
Source: J Clin Med. 2021 Dec 23;11(1):56. doi: 10.3390/jcm11010056 (PMC8745630; doi:10.3390/jcm11010056)
Supplement: Supplementary file 1 [file jcm-11-00056-s001.zip › jcm-1497169-supplementary.pdf]

Supplementary Material

Table S1. Univariate correlations between sST2 and selected variables in the total sample, in patients with stable COPD and exacerbated COPD.

	Total sample		Stable COPD		AE COPD	
	coef.	p-value	coef.	p-value	coef.	p-value
**Age, years**	0.097	0.296	0.086	0.528	0.044	0.830
**BMI, kg/m2**	-0.152	0.101	-0.040	0.772	-0.467	0.016
**Heart rate, bpm**	0.224	0.016	0.124	0.366	0.101	0.638
**Systolic BP, mmHg**	0.210	0.023	-0.421	0.001	-0.252	0.25
**Diastolic BP, mmHg**	0.078	0.403	0.071	0.606	-0.092	0.660
**Pack-years**	0.367	0.000	0.126	0.347	0.013	0.951
**FEV1, %predicted**	-0.417	0.000	-0.250	0.063	-0.588	0.002
**TLC, L**	0.413	0.000	0.207	0.146	0.262	0.226
**RV_TLC, ratio**	0.355	0.000	0.165	0.248	0.380	0.070
**6MWT, meters**	0.450	0.000	-0.466	0.001	0.066	0.614
**Neutrophils, G/L**	0.321	0.000	0.290	0.034	0.155	0.449
**CRP, mg/L**	0.261	0.000	-0.055	0.687	0.305	0.130
**Creatinine, mg/dl**	0.112	0.228	-0.036	0.794	0.099	0.630
**BUN, U/L**	0.032	0.729	-0.377	0.012	-0.127	0.537
**LDH, U/L**	0.300	0.001	0.149	0.278	0.400	0.052
**Cholesterol, mg/dl**	-0.208	0.024	-0.261	0.054	-0.201	0.303
**Triglyceride, mg/dl**	0.027	0.771	-0.087	0.529	-0.330	0.099
**HbA1c, %**	0.097	0.314	0.110	0.433	-0.202	0.393

Abbreviations: COPD, chronic obstructive pulmonary disease; AE COPD, acute exacerbation of chronic obstructive pulmonary disease; BMI, body mass index; HR, heart rate; BP, blood pressure; FEV1,% pred., forced expiratory volume in 1 second percentage predicted; TLC, total lung capacity; RV/TLC ratio, residual volume/total lung capacity; 6MWT, 6 minute walk test; CRP, C-reactive protein; BUN, blood urea nitrogen; LDH, lactate dehydrogenase; HbA1c, glycated hemoglobin.
